# “Skeletal Muscle Function Deficit” in A Nationally Representative British Birth Cohort in Early Old Age

**DOI:** 10.1093/gerona/glu214

**Published:** 2014-11-27

**Authors:** Rachel Cooper, David Bann, Elizabeth G. Wloch, Judith E. Adams, Diana Kuh

**Affiliations:** ^1^MRC Unit for Lifelong Health and Ageing, University College London, London, UK.; ^2^Clinical Radiology and Academic Health Science Centre, Manchester Royal Infirmary, Central Manchester University Hospital NHS Foundation Trust, Manchester, UK.

**Keywords:** Epidemiology, Life course, Physical capability.

## Abstract

**Background.:**

Recommendations for identifying age-related muscle dysfunction have recently been published. We aimed to compare definitions for clinically relevant weakness and low lean mass proposed by the Foundation for the National Institutes of Health (FNIH) Sarcopenia project with the definition of sarcopenia proposed by the European Working Group on Sarcopenia in Older People (EWGSOP).

**Methods.:**

A total of 1566 men and women from a British birth cohort had measures of appendicular lean mass, grip strength and timed up, and go speed at ages 60–64. Prevalence of low lean mass, weakness and slowness, identified using the FNIH and EWGSOP recommendations were estimated and compared: using kappa statistics and; by testing cross-sectional associations of both definitions of low lean mass and weakness with slowness and self-reported difficulties walking.

**Results.:**

The combined prevalence of low lean mass and weakness ranged from 1.1% in men using FNIH criteria to 6.4% in women using EWGSOP criteria. There was limited overlap between the groups identified using the different criteria, driven by limited agreement between the two definitions of low lean mass. Using FNIH criteria, both low lean mass and weakness were associated with higher odds of slowness and difficulties walking; whereas low lean mass classified using EWGSOP criteria was not associated with these markers of mobility impairment.

**Conclusions.:**

At relatively young ages, signs of skeletal muscle function deficit with potential clinical relevance are already identifiable in the general population. This suggests that implementation of strategies to prevent mobility limitations, related to age-related muscle dysfunction, may need to start at least as early as midlife.

A series of articles recently published outline the findings and recommendations of the Foundation for the National Institutes of Health (FNIH) Sarcopenia Project (1–5). This positive initiative helps to address some of the key challenges of research on sarcopenia and age-related muscle dysfunction which include: (a) identifying clinically meaningful age-related changes in muscle, and the factors across life that drive these changes and; (b) identifying modifiable targets for intervention which may reduce the detrimental impact of these changes on functional and clinical outcomes. If these and other initiatives result in more widely agreed operational definitions for what has recently been termed ‘‘skeletal muscle function deficit’’ (6), this may offer greater opportunities for evidence synthesis.

In direct response to the call made to evaluate the FNIH recommendations in other study populations (1,4,5), we aimed to use data from a nationally representative British birth cohort study (7), to compare the definitions for clinically relevant weakness and low lean mass proposed by the FNIH (2,3) with the definition of sarcopenia proposed by the European Working Group on Sarcopenia in Older People (EWGSOP) (8) which is now being applied in a wide range of settings (9–11). As mobility impairment has been identified as an important primary outcome (1,4) this comparison included tests of the associations of low lean mass and weakness with slow walking speed and self-reported difficulties walking.

## Methods

### Study Population

The Medical Research Council National Survey of Health and Development (NSHD) is a socially stratified sample of 5362 singleton births that took place in one week of March 1946 in mainland Britain. Between 2006 and 2010 (at 60–64 years), 2856 eligible study members (those known to be alive and living in England, Scotland, or Wales) were invited for assessment at one of six clinical research facilities (CRFs) or to be visited by a research nurse at home; 2229 were assessed (1690 at a CRF) (7). Relevant ethical approval was obtained and participants provided written consent.

### Measures At 60–64 Years

As described in detail elsewhere (12,13), measures of body composition (including appendicular lean mass [ALM] [the sum of the fat free mass in the limbs excluding bone mineral content]) were obtained for study participants who attended a CRF, in the supine position using a QDR 4500 Discovery DXA scanner (Hologic Inc, Bedford, MA).

Strength and physical performance were assessed by trained nurses during the CRF or home visit using standardized protocols. Grip strength (kg) was assessed using an electronic handgrip dynamometer (14). Three values were recorded for each hand; then the highest was used in analyses. Walking speed was assessed using the timed-up and go (TUG) test, with participants instructed to rise from a chair, walk 3 m at a normal pace, turn around, return to the chair, and sit down. The time taken to complete the test was recorded and the speed (m/s) calculated. Participants were also asked whether they had difficulty walking for quarter of a mile on the level because of long-term health problems. Height and weight were measured and used to calculate body mass index (BMI;kg/m^2^).

### Analyses

After deriving the variables required (ie ALM adjusted for height^2^, ALM:BMI ratio, and walking speed, by multiplying TUG speed by 1.62, based on comparisons of TUG and walking speeds in other British study populations with both measures [15]), the FNIH and EWGSOP cut-points for low lean mass, weakness and slowness were applied. Those participants in the potential analytic sample, who were unable to complete the grip strength (*n* = 17) or TUG (*n* = 8) assessment for health reasons (which included arthritis, other musculoskeletal conditions, and cardiorespiratory problems), were categorised as weak and slow, respectively.

Prevalence estimates of low lean mass, weakness, slowness, and the combinations of these outcomes were calculated. Cohen’s kappa was used to compare the different definitions. Logistic regression was used to estimate the odds ratios of slowness and self-reported difficulty walking associated with the different definitions of low lean mass and weakness. Gender-specific analyses were conducted on the sample with complete data on lean mass, grip strength, and walking speed (*n* = 1566). Sensitivity analyses, conducted to test the impact of including those people unable to perform the grip strength and TUG assessments in the main analyses and to assess the robustness of findings, are described in Appendix 1.

## Results

To aid comparison with similar estimates in other studies, baseline characteristics of the sample included in analyses are presented in Appendix 2. Prevalence estimates of low lean mass, weakness and slowness, and the combinations of these outcomes are shown in Table 1. Estimates of each of these three outcomes alone were much higher than combinations of these outcomes, reflecting limited overlap (see Appendix 3). As the FNIH criteria are more restrictive (5), prevalence estimates using these criteria were slightly lower than those using EWGSOP criteria.

**Table 1.** Prevalence Estimates (%) of Low Lean Mass, Weakness and Slowness Identified Using FNIH (1) and EWGSOP (8) Criteria (*n* = 747 men, 819 women)

	FNIH		EWGSOP	
	Men	Women	Men	Women
Low lean mass	15.7	14.9	20.8	30.7
Weakness	4.0	6.4	7.5	18.2
Slowness	9.0	9.7	9.0	9.7
Weakness and low lean mass	1.1	2.0	2.3	6.4
(Slowness or weakness) and low lean mass	3.1	2.7	4.4	7.3
Slowness and weakness and low lean mass	0.3	0.7	0	0.9

Cut-points:

Low lean mass (applied to ALM:BMI ratio for FNIH and ALM/ht^2^ for EWGSOP):

FNIH: Men < 0.789; women < 0.512.

EWGSOP: Men ≤ 7.23kg/m^2^; women ≤ 5.67kg/m^2^.

Weakness (applied to grip strength):

FNIH: Men < 26kg; women < 16kg.

EWGSOP: Men < 30kg; women < 20kg.

Slowness (applied to TUG speed after conversion to walking speed):

FNIH and EWGSOP: ≤0.8 m/s.

ALM, appendicular lean mass; BMI, body mass index; EWGSOP, European Working Group on Sarcopenia in Older People; FNIH, Foundation for the National Institutes of Health; TUG, timed-up and go test.

There was limited overlap between the groups identified as having both low lean mass and weakness using the different criteria (Ҡ = .23 in men, .09 in women), driven by limited agreement between the two definitions of low lean mass (Ҡ = .10 in men, .02 in women); 31.6% of men and 40.5% of women had low lean mass according to at least one definition, but only 4.8% of men and 5.0% of women were identified by both definitions.

Using the FNIH criteria, low lean mass and weakness were both associated with higher odds of slowness and self-reported difficulties walking (Table 2). Low lean mass classified using the EWGSOP criteria was not associated with mobility limitation.

**Table 2.** Odds Ratios of Slowness and Self-reported Difficulties Walking by Low Lean Mass and Weakness Identified Using FNIH and EWGSOP Criteria

	Odds ratio (95% CI) of:	
	Slowness*	Difficulty Walking^†^
Low lean mass		
FNIH		
Men	2.35 (1.33, 4.17)	3.44 (1.93, 6.11)
Women	1.80 (1.03, 3.18)	2.59 (1.48, 4.53)
EWGSOP		
Men	1.22 (0.68, 2.21)	0.77 (0.38, 1.55)
Women	0.86 (0.51, 1.44)	0.61 (0.34, 1.10)
Weakness		
FNIH		
Men	4.87 (2.13, 11.12)	4.73 (2.01, 11.16)
Women	3.13 (1.57, 6.26)	5.34 (2.76, 10.33)
EWGSOP		
Men	3.57 (1.81, 7.04)	2.84 (1.35, 5.97)
Women	2.63 (1.59, 4.35)	3.10 (1.84, 5.24)
Low lean mass and weakness		
FNIH		
Men	3.46 (0.68, 17.47)	7.30 (1.70, 31.32)
Women	6.00 (2.12, 16.98)	15.88 (5.71, 44.13)
EWGSOP		
Men	—	0.72 (0.09, 5.54)
Women	1.50 (0.65, 3.45)	1.81 (0.78, 4.18)

See Table 1 for definitions of low lean mass, weakness and slowness.

*n = 747 men, 819 women.

^†^ *n* = 745 men, 817 women.

FNIH, Foundation for the National Institutes of Health; EWGSOP, Eur opean Working Group on Sarcopenia in Older People.

Findings from sensitivity analyses suggested that there was no major impact on findings of including those people unable to complete the grip strength and TUG assessments for health reasons (Appendix 1).

## Discussion

In a nationally representative British birth cohort study, assessed in early old age, between 2.7% and 7.3% of the population were identified as having both low lean mass and either weakness or slowness according to two sets of recently published criteria (FNIH: 3.1% of men, 2.7% of women; EWGSOP: 4.4% of men, 7.3% of women) (1,8). Both definitions of weakness and the FNIH definition of low lean mass were strongly associated with slowness and self-reported difficulties walking. Despite producing lower prevalence estimates of low lean mass and weakness, the FNIH definitions were more strongly associated with mobility limitations than the EWGSOP definitions.

These analyses respond to the call to evaluate recently proposed criteria for the identification of clinically meaningful age-related changes in muscle mass, strength, and performance in other study populations (1,4,5). While the focus of existing research has been on populations aged 65 years and older (often with a mean age of 70 or older), our analyses of the NSHD at ages 60–64 highlights that at relatively young ages, signs of skeletal muscle function deficit with potential clinical relevance are already present in the general population; reflected in the finding of strong associations of weakness and low lean mass with increased odds of mobility limitation. This suggests that there may be opportunities for effective intervention at least as early as midlife to prevent mobility limitations that are related to age-related muscle dysfunction.

That some people in a general population in early old age already fell below cut-points for low lean mass, weakness and slowness may be due to initial developmental differences as well as earlier onset or accelerated rates of age-related decline in some individuals. Future studies, ideally with longitudinal assessment of muscle from earlier in life, should investigate these different pathways which may be associated with different health and disability prospects.

That the FNIH definition of low lean mass (which includes adjustment for BMI) was associated with slowness and difficulties walking; whereas, the EWGSOP definition of low lean mass (which includes adjustment for height) was not provided further evidence of the need to take account of BMI or fat mass when identifying those most likely to have insufficient muscle to support movement. As the FNIH definition uses a ratio of ALM to BMI, follow-up work is required to identify whether interventions targeting ALM, fat mass, or both are most effective in preventing future mobility disability.

This study has a number of major strengths as well as limitations which have recently been discussed in detail elsewhere (12). For example, our study population was selected to be nationally representative at baseline and remains so in many respects (16). However, our main analyses were restricted to those with complete data on all three outcomes. As highlighted previously (12), measures of lean mass were only attained for those people who underwent a DXA assessment during a CRF visit and, as also seen in most other studies which include detailed clinical assessments of muscle, these participants were in better health and less obese than those who were visited at home. Our sensitivity analyses (see Appendix 1) suggest that this may have led to underestimates of the prevalence of low lean mass, weakness and slowness suggesting that the need for earlier intervention may be even greater than our main results suggest.

Lean mass and walking speed were not assessed in the NSHD until the most recent data collection, and so we are currently limited to cross-sectional analyses of their associations with other outcomes alongside our prospective studies of their determinants (12,13). However, repeated measures of functional and clinical outcomes planned for future waves of data collection will facilitate longitudinal analysis of the consequences of skeletal muscle function deficit.

## Supplementary Material

Supplementary material can be found at: http://biomedgerontology.oxfordjournals.org/

## Funding

This work was supported by the UK Medical Research Council (Programme code MC_UU_12019/4). The study sponsors played no role in study design, the collection, analysis, and interpretation of data, the writing of the article, or the decision to submit it for publication.

## Conflicts of interest

None.

## Supplementary Material

Supplementary Data

## References

[1] Studenski SA Peters KW Alley DE The FNIH sarcopenia project: Rationale, study description, conference recommendations, and final estimates. J Gerontol A Biol Sci Med Sci. 2014;69:547–558. 24737557 10.1093/gerona/glu010 PMC3991146

[2] Alley DE Shardell MD Peters KW Grip strength cutpoints for the identification of clinically relevant weakness. J Gerontol A Biol Sci Med Sci. 2014;69:559–566. 24737558 10.1093/gerona/glu011 PMC3991145

[3] Cawthon PM Peters KW Shardell MD Cutpoints for low appendicular lean mass that identify older adults with clinically significant weakness. J Gerontol A Biol Sci Med Sci. 2014;69:567–575. 24737559 10.1093/gerona/glu023 PMC3991141

[4] McLean RR Shardell MD Alley DE Criteria for clinically relevant weakness and low lean mass and their longitudinal association with incident mobility impairment and mortality: the foundation for the National Institutes of Health (FNIH) sarcopenia project. J Gerontol A Biol Sci Med Sci. 2014;69:576–583. 24737560 10.1093/gerona/glu012 PMC3991140

[5] Dam TT Peters KW Fragala M An evidence-based comparison of operational criteria for the presence of sarcopenia. J Gerontol A Biol Sci Med Sci. 2014;69:584–590. 24737561 10.1093/gerona/glu013 PMC3991139

[6] Correa-de-Araujo R Hadley E Skeletal muscle function deficit: A new terminology to embrace the evolving concepts of sarcopenia and age-related muscle dysfunction. J Gerontol A Biol Sci Med Sci. 2014;69:591–594. 24737562 10.1093/gerona/glt208 PMC3999854

[7] Kuh D Pierce M Adams J Cohort profile: Updating the cohort profile for the MRC National Survey of Health and Development: A new clinic-based data collection for ageing research. Int J Epidemiol. 2011;40:e1–e9. 21345808 10.1093/ije/dyq231 PMC3043283

[8] Cruz-Jentoft AJ Baeyens JP Bauer JM Sarcopenia: European consensus on definition and diagnosis: Report of the European Working Group on Sarcopenia in Older People. Age Ageing. 2010;39:412–423. 20392703 10.1093/ageing/afq034 PMC2886201

[9] Volpato S Bianchi L Cherubini A Prevalence and clinical correlates of sarcopenia in community-dwelling older people: application of the EWGSOP definition and diagnostic algorithm. J Gerontol A Biol Sci Med Sci. 2014;69:438–446. 24085400 10.1093/gerona/glt149 PMC3968828

[10] Patel HP Syddall HE Jameson K Prevalence of sarcopenia in community-dwelling older people in the UK using the European Working Group on Sarcopenia in Older People (EWGSOP) definition: findings from the Hertfordshire Cohort Study (HCS). Age Ageing. 2013;42:378–384. 23384705 10.1093/ageing/afs197 PMC3633365

[11] Vetrano DL Landi F Volpato S Association of sarcopenia with short- and long-term mortality in older adults admitted to acute care wards: Results from the CRIME study. J Gerontol A Biol Sci Med Sci. 2014;69:1154–1161. 24744390 10.1093/gerona/glu034

[12] Cooper R Hardy R Bann D Body mass index from age 15 years onwards and muscle mass, strength, and quality in early old age: Findings from the MRC National Survey of Health and Development. J Gerontol A Biol Sci Med Sci. 2014;69:1253–1259. 24682351 10.1093/gerona/glu039 PMC4158414

[13] Bann D Cooper R Wills AK Adams J Kuh D; NSHD scientific and data collection team. Socioeconomic position across life and body composition in early old age: Findings from a British birth cohort study. J Epidemiol Community Health. 2014;68:516–523. 24567442 10.1136/jech-2013-203373 PMC4033171

[14] Kuh D Hardy R Butterworth S Developmental origins of midlife grip strength: findings from a birth cohort study. J Gerontol A Biol Sci Med Sci. 2006;61:702–706. 16870632 10.1093/gerona/61.7.702

[15] Cooper R Hardy R Aihie Sayer A Age and gender differences in physical capability levels from mid-life onwards: The harmonisation and meta-analysis of data from eight UK cohort studies. PLoS One. 2011;6:e27899. 22114723 10.1371/journal.pone.0027899 PMC3218057

[16] Stafford M Black S Shah I Using a birth cohort to study ageing: representativeness and response rates in the National Survey of Health and Development. Eur J Ageing. 2013;10:145–147. 23637643 10.1007/s10433-013-0258-8 PMC3637651

