# Supplementary material for: “Skeletal Muscle Function Deficit” in A Nationally Representative British Birth Cohort in Early Old Age
Source: J Gerontol A Biol Sci Med Sci. 2014 Nov 27;70(5):604–7. doi: 10.1093/gerona/glu214 (PMC4386990; doi:10.1093/gerona/glu214)
Supplement: Supplementary Data [file supp_70_5_604__index.html]

“Skeletal Muscle Function Deficit” in A Nationally Representative British Birth Cohort in Early Old Age — “Skeletal Muscle Function Deficit” in A Nationally Representative British Birth Cohort in Early Old Age — Supplementary Data 

# “Skeletal Muscle Function Deficit” in A Nationally Representative British Birth Cohort in Early Old Age

## Supplementary Data

Data files

**Files in this Data Supplement:**

- Supplementary Data - Supplementary Data
