# Supplementary material for: “Skeletal Muscle Function Deficit” in A Nationally Representative British Birth Cohort in Early Old Age
Source: J Gerontol A Biol Sci Med Sci. 2014 Nov 27;70(5):604–7. doi: 10.1093/gerona/glu214 (PMC4386990; doi:10.1093/gerona/glu214)

**Supplementary information**

**Appendix 1: Sensitivity analyses**

A series of sensitivity analyses were performed in which: (1) those people who were unable to perform the grip strength and timed up and go assessments for health reasons (n=17 and 8, respectively) were excluded from prevalence estimates (see table a)and also the analyses of the associations of low lean mass and weakness with slowness and self-reported difficulties walking (see table b); (2) prevalence estimates of low lean mass, weakness and slowness were estimated using the maximum available samples for each measure (rather than restricting analyses to the sample with complete data on all three measures) (see table c); (3) in analyses of the associations of low lean mass and weakness with self-reported difficulty walking, stricter criteria were employed to identify those with self-reported walking difficulties by taking into account a second question which asked those who reported difficulty walking for quarter of a mile on the level because of long-term health problems how far they could walk without stopping or severe discomfort (response options: more than 400 yards; 200-400 yards; 50-200 yards; less than 50 yards) with those who reported difficulty but that they could walk more than 400 yards grouped with those who reported no difficulty (see table d).

**Table a: Prevalence estimates of low lean mass, weakness and slowness identified using FNIH and EWSGOP criteria, with exclusion of those people unable to perform the grip strength and/or TUG assessments for health reasons (N=737 men, 807 women)**

|  | **FNIH** | | **EWSGOP** | |
| --- | --- | --- | --- | --- |
|  | **Men** | **Women** | **Men** | **Women** |
| **Low lean mass** | 15.5 | 14.9 | 20.6 | 30.7 |
| **Weakness** | 3.3 | 5.1 | 6.8 | 17.0 |
| **Slowness** | 8.3 | 9.2 | 8.3 | 9.2 |

Footnote: In the main analytical sample of 1566,participants who were unable to complete the grip strength (n=17 (6 men, 11 women)) and TUG (n=8 (6 men, 2 women)) assessments for health reasons were classified as weak and slow, respectively. The main reason reported for being unable to complete the grip strength assessment was arthritis (reported by 41% of those unable) followed by other musculoskeletal conditions. Reasons for not completing the TUG assessment included joint problems and cardio-respiratory symptoms.

**Table b: Odds ratios of slowness and self-reported difficulties walking by low lean mass and weakness identified using FNIH and EWGSOP criteria, with exclusion of those people unable to perform the grip strength and/or TUG assessments for health reasons**

|  | **Odds ratio (95% CI) of:** | |
| --- | --- | --- |
|  | **slowness** | **difficulty walking** |
| **Low lean mass**  FNIH: men  women  EWGSOP: men  women | 2.49 (1.38, 4.49)^*^  1.73 (0.97, 3.09)^*^  1.27 (0.69, 2.34)^*^  0.84 (0.49, 1.41)^*^ | Same as main model |
| **Weakness**  FNIH: men  women  EWGSOP: men  women | 5.03 (2.00, 12.65)^†^  2.57 (1.14, 5.79) ^†^  3.59 (1.73, 7.44) ^†^  2.27 (1.33, 3.89) ^†^ | 3.43 (1.23, 9.55)^±^  4.25 (1.98, 9.12)^±^  2.13 (0.91, 4.97)^±^  2.64 (1.51, 4.61)^±^ |
| **Low lean mass and weakness** | |  |
| FNIH: men  women  EWGSOP: men  women | 5.70 (1.02, 31.74) ^†^  4.13 (1.26, 13.51) ^†^  -  1.42 (0.58, 3.45) ^†^ | 6.29 (1.13, 35.10)^±^  12.89 (4.37, 38.03)^±^  0.87 (0.11, 6.73)^±^  2.05 (0.88, 4.76)^±^ |

* N (after excluding those unable to perform the TUG assessment): men=741, women=817

^†^ N (after excluding those unable to perform the TUG and/or grip strength assessment): men=737, women=807

± N (after excluding those unable to perform the grip strength assessment): men=739, women=806

**Table c: Prevalence estimates of low lean mass, weakness and slowness identified using FNIH and EWSGOP criteria, using the maximum available samples for each measure**

|  | **N** | | **FNIH** | | **EWSGOP** | |
| --- | --- | --- | --- | --- | --- | --- |
|  | **Men** | **Women** | **Men** | **Women** | **Men** | **Women** |
| **Low lean mass** | 769 | 842 | 16.0 | 15.0 | 20.4 | 31.2 |
| **Weakness** | 1018 | 1100 | 6.1 | 9.9 | 10.4 | 21.5 |
| **Slowness** | 982 | 1082 | 10.8 | 13.3 | 10.8 | 13.3 |

**Table d: Odds ratios of self-reported difficulties walking by low leanmass and weakness identified using FNIH and EWGSOP criteria**

|  | **Odds ratio (95% CI) of:** |
| --- | --- |
|  | **difficulty walking^*^** |
| **Low leanmass**  FNIH: men  women  EWGSOP: men  women | 4.15 (2.16, 7.96)  2.76 (1.48, 5.15)  0.90 (0.41, 1.98)  0.74 (0.39, 1.42) |
| **Weakness**  FNIH: men  women  EWGSOP: men  women | 5.91 (2.38, 14.71)  3.51 (1.61, 7.68)  3.81 (1.72, 8.42)  2.84 (1.57, 5.15) |
| **Low leanmass and weakness** | |
| FNIH: men  women  EWGSOP: men  women | 5.81 (1.14, 29.70)  13.07 (4.65, 36.69)  1.05 (0.14, 8.09)  1.66 (0.63, 4.38) |

* Self-reported difficulty walking less than 400 yards on the level (5.6% in men and 6.4% in women)

**Appendix 2:**

**Table a: Characteristics of the MRC National Survey of Health and Development at ages 60-64 years [sample restricted to those with data on grip strength, appendicular lean mass and walking speed (N=1566 (747 Men, 819 Women))]**

|  | **Mean (SD)** | |
| --- | --- | --- |
|  | **Men** | **Women** |
| **Age (y)** | 63.2 (1.2) | 63.3 (1.1) |
| **Weight (kg)** | 85.3 (13.0) | 72.2 (13.6) |
| **Height (m)** | 1.8 (0.1) | 1.6 (0.1) |
| **BMI (kg/m^2^)** | 27.7 (3.9) | 27.5 (5.0) |
| **TUG speed (m/s)** | 0.7 (0.2) | 0.7 (0.1) |
| **TUG rescaled* (m/s)** | 1.2 (0.3) | 1.1 (0.2) |
| **Grip strength (kg)** | 46.9 (11.4) | 27.2 (7.3) |
| **ALM (kg)** | 24.6 (3.4) | 16.2 (2.5) |
| **ALM/ht^2^ (kg/m^2^)** | 8.0 (0.9) | 6.2 (0.9) |
| **ALM:BMI ratio** | 0.9 (0.1) | 0.6 (0.1) |
| **Self-reported difficulties walking ¼ mile (%)** | 7.9 | 8.5 |
| **Clinical disorders (%)^†^** |  |  |
| **Obesity** | 26.6 | 27.4 |
| **Cardiovascular disease** | 12.4 | 5.9 |
| **Hypertension** | 35.8 | 27.1 |
| **Diabetes** | 12.5 | 6.7 |
| **Cancer** | 9.3 | 13.8 |
| **Osteoporosis** | 5.6 | 14.7 |

* Multiplied by factor of 1.62

† For details of ascertainment and definitions used please see: Pierce MB, Silverwood RJ, Nitsch D et al. Clinical disorders in a post war British cohort reaching retirement: Evidence from the first national birth cohort study. *PLoS One* 2012;7(9):e44857. Ns vary due to missing data on clinical disorders.

BMI = body mass index

TUG = Timed up and go test

ALM = appendicular lean mass

**Table b: Characteristics of the MRC National Survey of Health and Development at ages 60-64 years stratified by prevalent FNIH and EWGSOP criterion [sample restricted to those with data on grip strength, appendicular lean mass and walking speed (Maximum N=1566 (747 Men, 819 Women))]**

|  | **FNIH criterion** | | | |  | **EWGSOP criterion** | | | |
| --- | --- | --- | --- | --- | --- | --- | --- | --- | --- |
|  | **Men** | | **Women** | |  | **Men** | | **Women** | |
|  | **No**  **(n=722)** | **Yes^*^**  **(n=25)** | **No**  **(n=791)** | **Yes^*^**  **(n=28)** |  | **No**  **(n=714)** | **Yes^†^**  **(n=33)** | **No**  **(n=752)** | **Yes^†^**  **(n=67)** |
| **Weight (kg)** | 85.4 (12.9) | 82.9 (15.5) | 72.0 (13.4) | 81.3 (14.5) |  | 86.0 (12.7) | 69.8 (10.3) | 73.2 (13.5) | 61.4 (8.1) |
| **Height (m)** | 1.8 (0.1) | 1.7 (0.1) | 1.6 (0.1) | 1.6 (0.1) |  | 1.8 (0.1) | 1.7 (0.1) | 1.6 (0.1) | 1.6 (0.1) |
| **BMI (kg/m^2^)** | 27.6 (3.9) | 29.5 (4.5) | 27.3 (4.8) | 33.7 (5.4) |  | 27.9 (3.9) | 23.8 (2.8) | 27.8 (5.0) | 23.5 (2.8) |
| **TUG speed (m/s)** | 0.7 (0.2) | 0.5 (0.1) | 0.7 (0.1) | 0.5 (0.1) |  | 0.7 (0.2) | 0.6 (0.2) | 0.7 (0.1) | 0.6 (0.2) |
| **Grip strength (kg)** | 47.3 (11.1) | 34.4 (11.4) | 27.5 (7.1) | 17.5 (5.9) |  | 47.5 (11.1) | 33.9 (10.8) | 28.0 (7.0) | 18.8 (4.8) |
| **ALM/ht^2^ (kg/m^2^)** | 8.0 (0.9) | 7.5 (1.1) | 6.1 (0.8) | 6.7 (1.0) |  | 8.1 (0.9) | 6.5 (0.5) | 6.2 (0.8) | 5.2 (0.4) |
| **ALM:BMI ratio** | 0.9 (0.1) | 0.7 (0.1) | 0.6 (0.1) | 0.5 (0.03) |  | 0.9 (0.1) | 0.8 (0.1) | 0.6 (0.1) | 0.6 (0.1) |
| **Clinical disorders (%)^±^** |  |  |  |  |  |  |  |  |  |
| **Obesity** | 25.9 | 48.0 | 25.8 | 71.4 |  | 27.9 | 0.0 | 29.8 | 0.0 |
| **Cardiovascular disease** | 11.8 | 33.3 | 5.6 | 17.7 |  | 12.1 | 20.0 | 5.3 | 13.5 |
| **Hypertension** | 35.0 | 60.0 | 26.4 | 46.4 |  | 35.6 | 39.4 | 27.2 | 25.4 |
| **Diabetes** | 12.2 | 23.8 | 6.2 | 20.8 |  | 12.8 | 7.1 | 6.7 | 7.6 |
| **Cancer** | 9.3 | 8.7 | 13.6 | 20.8 |  | 9.4 | 6.9 | 13.5 | 17.5 |
| **Osteoporosis** | 5.7 | 4.0 | 14.8 | 14.3 |  | 5.2 | 15.2 | 13.4 | 30.3 |

* Classified as: (low lean mass and weakness and slowness) OR (low lean mass and (weakness or slowness)) according to FNIH criteria

† Classified as: (low lean mass and weakness and slowness) OR (low lean mass and (weakness or slowness)) according to EWGSOP criteria

± For details of ascertainment and definitions used please see: Pierce MB, Silverwood RJ, Nitsch D et al. Clinical disorders in a post war British cohort reaching retirement: Evidence from the first national birth cohort study. *PLoS One* 2012;7(9):e44857. Ns vary due to missing data on clinical disorders.

BMI = body mass index

TUG = Timed up and go test

ALM = appendicular lean mass

**Appendix 3: Independence and overlap of prevalent FNIH and EWGSOP criterion (low lean mass, weakness and slowness) among men (N=747) and women (N=819) in the MRC National Survey of Health and Development**

**A. FNIH criteria (Men) C. EWGSOP criteria (Men)**

1.7%

2.1%

2.3%

0%

0.9%

2.3%

0.8%

0.3%


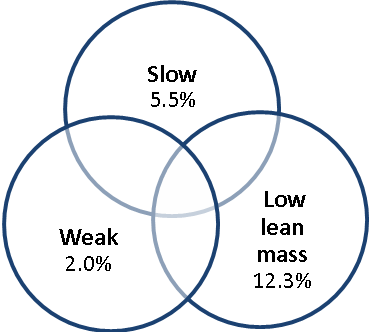

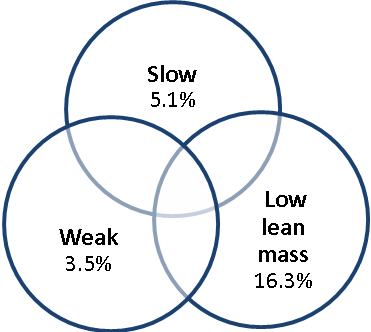


**B. FNIH criteria (Women) D. EWGSOP criteria (Women)**

5.5%

2.4%

1.8%

0.9%

0.7%

1.2%

0.7%

1.5%


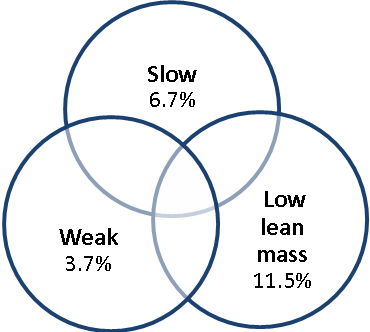

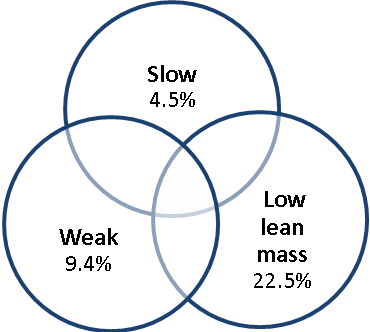

Supplement: Supplementary Data [file supp_glu214_Appendices_revised.docx]
